# Performing up to Nordic principles? Geographic and socioeconomic equity in ambulatory care sensitive conditions among older adults in capital areas of Denmark, Finland and Sweden in 2000–2015

**DOI:** 10.1186/s12913-023-09855-0

**Published:** 2023-08-07

**Authors:** Markku Satokangas, Martti Arffman, Janne Agerholm, Karsten Thielen, Charlotte Ørsted Hougaard, Ingelise Andersen, Bo Burström, Ilmo Keskimäki

**Affiliations:** 1 Health Economics and Equity in Health Care, Finnish Institute for Health and Welfare, P.O. Box 30, 00271 Helsinki, Finland; 2 Network of Academic Health Centres and Department of General Practice and Primary Health Care, University of Helsinki, P.O. Box 20, 00014 Helsinki, Finland; 3 Department of Global Public Health, Karolinska Institutet, Stockholm, Sweden; 4 Section of Social Medicine, Department of Public Health, University of Copenhagen, Copenhagen, Denmark; 5 Faculty of Social Sciences, Tampere University, 33014 Tampere, Finland

**Keywords:** Equity in health care, Primary health care, Geographic disparities, Socioeconomic disparities, Health service research, Register-based study, International comparison, Nordic countries

## Abstract

**Background:**

Denmark, Finland and Sweden pursue equity in health for their citizens through universal health care. However, it is unclear if these services reach the older adult population equally across different socioeconomic positions or living areas. Thus, we assessed geographic and socioeconomic equity in primary health care (PHC) performance among the older adults in the capital areas of Denmark (Copenhagen), Finland (Helsinki) and Sweden (Stockholm) in 2000–2015. Hospitalisations for ambulatory care sensitive conditions (ACSC) were applied as a proxy for PHC performance.

**Methods:**

We acquired individual level ACSCs for those aged ≥ 45 in 2000–2015 from national hospitalisation registers. To identify whether the disparities varied by age, we applied three age groups (those aged 45–64, 65–75 and ≥ 75). Socioeconomic disparities in ACSCs were described with incidence rate ratios (IRR) and annual rates by education, income and living-alone; and then analysed with biennial concentration indices by income. Geographic disparities were described with biennial ACSC rates by small areas and analysed with two-level Poisson multilevel models. These models provided small area estimates of IRRs of ACSCs in 2000 and their slopes for development over time, between which Pearson correlations were calculated within each capital area. Finally, these models were adjusted for income to distinguish between geographic and socioeconomic disparities.

**Results:**

Copenhagen had the highest IRR of ACSCs among those aged 45–64, and Helsinki among those aged ≥ 75. Over time IRRs decreased among those aged ≥ 45, but only in Helsinki among those aged ≥ 75. All concentration indices slightly favoured the affluent population but in Stockholm were mainly non-significant. Among those aged ≥ 75, Pearson correlations were low in Copenhagen (-0.14; *p* = 0.424) but high in both Helsinki (-0.74; < 0.001) and Stockholm (-0.62; < 0.001) – with only little change when adjusted for income. Among those aged ≥ 45 the respective correlations were rather similar, except for a strong correlation in Copenhagen (-0.51, 0.001) after income adjustment.

**Conclusions:**

While socioeconomic disparities in PHC performance persisted among older adults in the three Nordic capital areas, geographic disparities narrowed in both Helsinki and Stockholm but persisted in Copenhagen. Our findings suggest that the Danish PHC incorporated the negative effects of socio-economic segregation to a lesser degree.

**Supplementary Information:**

The online version contains supplementary material available at 10.1186/s12913-023-09855-0.

## Background

Denmark, Finland and Sweden share the challenge of arranging universal care for their ageing populations. Their primary health care (PHC) systems have different arrangements, but common principles of decentralised municipal and regional implementation with limited steering from national governments [1–4]. Although universal PHC reduces health inequities [5], it is unclear whether these services actually reach older adults equally between different socioeconomic positions (SEP) and living-areas. The main challenge for unravelling this is the lack of indicators for comprehensive PHC performance. Disease-specific indicators and results of single laboratory tests or clinical measurements rarely capture the diversity of PHC – in which comprehensive care usually means simultaneous management of several multimorbid conditions, avoidance of overtreatment, and answering to the needs of both each individual patient and the population as a whole [6–8].

Despite its limitations, hospitalisations for ambulatory care sensitive conditions (ACSCs) are currently one of the best available proxy indicators for comprehensive PHC performance [9, 10]. Indeed, it is endorsed by national stewards and international organisations as an indicator of performance (e.g. OECD analyses ACSC as a quality indicator in their ‘Health at a Glance’ publications [11], and some countries analyse ACSCs in their PHC evaluation processes). It captures conditions in which unmet health needs can lead to hospitalisations that are possibly preventable by PHC [9]. While only part of ACSCs seem to be truly preventable by PHC, the quality of general practitioner (GP) consultations and organisational aspects of PHC often emerge among the factors contributing to them [12, 13]. For example, a low level of ACSCs associates with good continuity of care in PHC [14, 15], a high supply of family physicians [16] and PHC payment models that reward comprehensive care [17].

On the other hand, ACSCs are also impacted by factors that either confound the measurement of PHC performance or are beyond the scope of PHC. Indeed, risk of ACSCs associates strongly with factors related to individual characteristics such as self-rated health [18], comorbidities [18–20] and SEP [18, 20, 21]. Older age especially predisposes people to ACSCs through the accumulation of comorbid conditions [22], their increased severity [23], and limitations in activities of daily living [24]. Thus, unsurprisingly, ACSCs mainly concentrate on the population over 60 years of age [18, 25–27]. Moreover, several other patient-level issues (such as adherence to treatment, self-management skills, mental health problems and engagement with health care) also contribute to ACSCs [13, 28]. To reduce ACSCs, earlier literature recommends improving not only vulnerable populations’ health care, but also their social and physical environment as well as their opportunities for transport [29, 30].

Overall, clear geographic [18, 26, 31–35] and socioeconomic disparities [21, 36, 37] in ACSCs are common findings in several regional and national studies. The socioeconomic disparities in ACSCs emerge as an inverse socioeconomic gradient that disfavours the poorest [21, 37], with the risk increasing even further due to accumulation and prolongation of different socioeconomic disadvantages over time [36]. While the geographic disparities in ACSCs can mirror variation in PHC quality [32], they also mirror the differences for example in inhabitants’ health status and SEP [18, 20, 33, 38] as well as structural differences related to areas’ hospital supply and arrangement of hospital care [20, 39].

Due to several reasons (e.g. the country-specific nature of ACSCs, its various definitions, and challenges in drawing useful lessons of its international comparisons), only few international comparisons exist of socioeconomic or geographic variations in ACSCs [40, 41]. These studies have analysed disparities in ACSCs across larger geographic regions and were adjusted for area-level SEP. Thus, a gap exists for the international comparison of disparities in ACSCs by small areas adjusted for individual SEP. Such a comparison could provide important insights into the different national strategies to organise PHC – unattainable by analyses of ACSCs within a single country – and with greater detail than the international comparisons of regional or country-level ACSCs. Also, no comparison has been made between Nordic countries, which have rather similar principles for providing health care and pursuing health equity. This study aims to fill these gaps in knowledge by analysing geographic and socioeconomic equity in ACSC between older adults living in the capital areas of Denmark, Finland, and Sweden. Moreover, to avoid over-interpreting direct international comparison of ACSCs (which can be misleading due to differences national care pathways or arrangement of hospital care, for example), we aimed to observe and compare the development of ACSCs by small areas over time in the three Nordic capital areas, and assess whether this development would be affected by individual SEP.

Thus, we asked the following questions in parallel and combined analyses in Copenhagen, Helsinki and Stockholm: 1) What level and development over time of ACSCs emerged among their older adult population? 2) How were these ACSCs distributed by individual age, SEP and small area of residence? 3) What level and development over time of socioeconomic disparities emerged in these ACSCs? 4) How did geographic disparities in these ACSCs develop over time? 5) Did this development of geographic disparities over time mirror geographic disparities in the population’s socioeconomic structure?

### Context in Denmark, Finland, and Sweden

Each of these three countries finance their PHC mainly by taxation, supplemented with out-of-pocket payments (highest in Finland and lowest in Denmark). Finnish GPs work in public health centres with a broad multidisciplinary staff, Swedish GPs work in a similar way but with smaller team-based units (both public and private), and Danish GPs work as publicly-funded private practitioners operating either alone or in small collaborative groups. In Finland, two alternative pathways to GP consultations also exist, which operate in parallel to but distinct from the public PHC: private health care and occupational health care. However, the clientele of these alternative pathways is limited consecutively by patients’ wealth or working status. Unlike in Sweden, both Finnish and Danish GPs act as gatekeepers to hospital care. Though some private hospitals exist, hospital care is mainly provided by public hospitals. Moreover, all three countries have steadily reduced their curative hospital bed supply over time: from 426 per 100,000 inhabitants in 2000 to 246 in 2015 in Denmark, from 393 to 305 in Finland and from 310 to 226 in Sweden [42]. While Denmark has no user fees for PHC or secondary care Finland and Sweden have limited fees for both (approximately €21–22 for PHC and €38–40 for secondary care). In recent decades, Sweden and Denmark have given the responsibility for organising care to larger administrative areas and have adopted a PHC purchaser-provider split.

## Methods

### Data

We examined ACSC hospitalisations for the population aged 45 years and over in the capital areas of Denmark (Copenhagen), Finland (Helsinki) and Sweden (Stockholm) in 2000–2015. Hospitalisation records were obtained from routinely collected registers: the Danish National Patient Register, the Finnish Care Register for Health Care and the Patient Administrative Register from Region Stockholm. These registers cover virtually all hospitalisations in both public and private hospitals within each of the capital areas. The Danish and Finnish registers include hospital discharges, while the Swedish register includes hospital admissions. We defined ACSCs in hospitalisations according to the UK definition of ACSCs [43] supplemented with unspecified pneumonia (ICD-10 diagnosis code J18.9) – as previously applied in Finland [44]. To maintain backward compatibility, we used both ICD-9 and ICD-10 codes. Moreover, we translated this definition to match the Swedish version of ICD-9 and both the Danish and Swedish versions of NOMESCO Classification of Surgical Procedures (Additional file 1). For each individual, we combined ACSC periods within one day of each other. This way, we accounted for any possible hospital transfers or immediate readmissions. Linkages between hospitalisation data and individual sociodemographic data were done using personalised ID codes.

For each individual in our study population, we were able to link data for age, sex, education, living circumstances, household income, municipality of residence and (in the case of larger municipalities) city district of residence. This data was obtained from registries maintained by Statistics Denmark, FOLK and INFRA databases by Statistics Finland, and the compound LISA dataset by Statistics Sweden. Altogether, there were 36 small areas in Copenhagen, 47 in Helsinki and 39 in Stockholm (Additional file 2).

Age was grouped into five-year age bands – and for the purposes of multilevel models additionally into three larger age groups (population aged 45–64, 65–74 and 75 and over). Education was grouped into primary, secondary, and tertiary education according to the International Standard Classification of Education levels. Household net income was grouped into income deciles according to decile limits derived from each respective national population. To prevent indirect identification of individuals, all small area data was grouped into biennial time periods. The population at risk of ACSCs was calculated as a mean resident population each year and within each capital area. These values were used as an estimate for person-years – their numbers by small areas ranged between 7,200–42,500 in Copenhagen, 1,900–24,300 in Helsinki and 4,200–54,900 in Stockholm in 2015.

### Statistical analysis

A direct method of standardisation was applied to calculate gender-standardised ACSC rates by education, income and living circumstances as well as age-standardised and gender-standardised ACSC rates by small areas. The European Standard Population (2013) was used as the standard population [45]. ACSC hospitalisations were also analysed separately in each of the capital areas by univariate Poisson models to obtain incidence rate ratios (IRRs) – which were then used to predict ACSCs. Moreover, biennial concentration indices (Cs) were calculated in eight time-periods and the three capital areas by ranking age-standardised ACSC rates by income deciles. This approach has been described in more detail in previous literature [46–48]. Cs are a measure of health disparities by income, interpreted against the value of 0 (which a represents situation with no disparities). Cs of -1 would represent absolute disparities with all ACSCs emerging in the lowest income decile, and that of 1, absolute disparities with all ACSCs in the highest income decile.

Finally, we were able to combine data from the three capital areas and estimate the development of geographic disparities over time using two-level Poisson multilevel models with population at risk applied as an offset: individuals nested in 122 small areas with the capital area of each individual included as a fixed effect. Separate models were built for the total study population (Models 1 and 2) and for those aged 75 and over (Models 3 and 4). Each model was adjusted for gender and biennial time periods, and included interactions with the effect of capital area. Models 1 and 2 were also adjusted for age group and Models 2 and 4 additionally with individual incomes. As with the univariate models, IRRs were also calculated from the multilevel models. For estimates with interactions, selected contrasts were both defined and tested with a general linear hypothesis test. Moreover, these models provided estimates for both small area intercepts for ACSCs and their slopes for development over time, between which Pearson correlations were calculated. The statistical analyses were performed with R, release versions 4.0.2 and 4.0.4 [49] – with the Laplace approximation and packages lme4 [50] and multcomp [51].

## Results

**Fig. 1:**
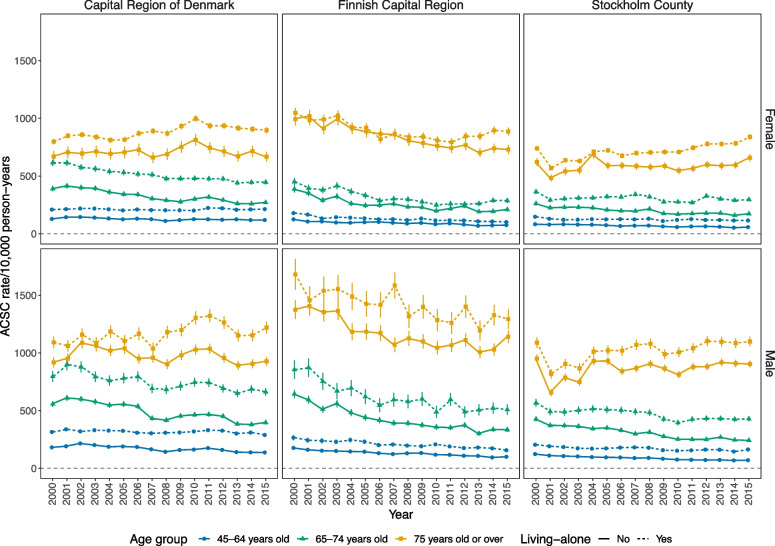
ACSC rates by living circumstance Age-standardised rates of hospitalisations for ambulatory care sensitive conditions (ACSC) among those living alone in three Nordic capital areas in 2000–2015

Overall, the total study population in the capital areas of Denmark (Copenhagen) and Sweden (Stockholm) were nearly twice of the capital area of Finland (Helsinki) in 2000–2015 (Table 1). Moreover, while the age-standardised and gender-standardised ACSC rates per 10,000 person years somewhat decreased in each of the capital areas, this decrease seemed to be the most pronounced in Helsinki (Table 2 & Fig. 1). In univariate models analysed separately within each capital area, ACSCs were on average more common in the older age groups in 2000–2015, being 2.63 to 2.96-fold in those aged 65–74 years, and 4.54 to 8.11-fold in 75 years and over, in the three capitals. Moreover, male gender, living-alone, lower income and lower education predicted higher incidence of ACSCs in each capital area and in each age group (Table 3).

**Table 1:** Characteristics of the study population in three Nordic capital areas in 2000 and 2015

	Copenhagen		Helsinki		Stockholm	
Year	2000	2015	2000	2015	2000	2015
Total study population (n)	626,931	788,263	342,503	442,623	715,305	897,841
Gender (%)						
Female	54.3	52.2	57.3	55.4	53.7	52.0
Male	45.7	47.8	42.7	44.6	46.3	48.0
Age group (%)						
45–49	17.5	19.0	19.5	16.7	16.2	17.3
50–54	18.0	17.8	21.9	16.6	17.7	16.2
55–59	16.1	14.6	15.7	14.6	16.8	13.8
60–64	11.8	12.2	11.7	13.5	11.4	12.3
65–69	9.7	11.9	9.4	13.9	9.0	12.5
70–74	8.7	9.8	8.1	9.4	8.5	10.6
75–79	7.8	6.4	6.2	6.6	8.3	6.8
80–84	5.5	4.2	4.0	4.6	6.3	4.7
85 +	4.9	4.2	3.4	4.2	5.8	5.8
Living alone (%)						
No	59.7	60.2	70.5	68.5	61.0	62.0
Yes	40.3	39.8	29.5	31.5	39.0	38.0
Education (%)						
Tertiary	22.7	35.2	33.3	44.4	26.3	33.6
Secondary	35.3	39.7	24.0	28.2	32.8	46.5
Primary	28.9	21.9	42.7	27.4	20.6	18.2
NA	13.1	3.2	0	0	20.2	1.6
Income quintile (%)						
Highest	30.8	28.3	37.6	36.1	22.7	25.8
4	16.7	17.9	19.5	19.7	21.5	20.2
3	13.8	16.2	15.6	15.9	20.3	18.9
2	17.3	17.5	15.5	14.0	19.8	19.2
Lowest	18.5	17.9	9.6	12.5	12.7	13.2
NA	3.0	2.2	2.3	1.8	2.9	2.7

**Table 2:** Incidence of ACSCs in 2000–2015

	Copenhagen	Helsinki	Stockholm
Number of ACSCs in 2000–2015	394,793	180,227	335,357
ACSC rate in 2000	410 (405–416)	464 (455–474)	330 (325–334)
ACSC rate in 2015	377 (372–382)	323 (316–329)	284 (280–288)
Change in ACSC rates from 2000 to 2015			
in total study population	-8%	-30%	-14%
by three age groups			
45–64	-9%	-38%	-30%
65–74	-27%	-49%	-31%
75 and over	+ 6%	-25%	+ 2%

Incidence of hospitalisations for ambulatory care sensitive conditions (ACSCs) separately in three Nordic capital areas in 2000–2015: number of ACSCs as well as age-standardised and gender-standardised ACSC rates by 10,000 person years

**Table 3:** IRRs of individual factors associated with ACSCs in 2000–2015, univariate Poisson models

Age group	Variable	Copenhagen	Helsinki	Stockholm
45–64	Gender			
	Female	1	1	1
	Male	1.34 (1.33–1.35)	1.41 (1.39–1.44)	1.36 (1.35–1.38)
	Living alone			
	No	1	1	1
	Yes	1.74 (1.72–1.75)	1.49 (1.46–1.52)	1.99 (1.96–2.02)
	Education			
	Tertiary	1	1	1
	Secondary	1.78 (1.76–1.81)	1.69 (1.65–1.73)	1.73 (1.70–1.77)
	Primary	2.99 (2.95–3.03)	2.72 (2.67–2.78)	2.90 (2.84–2.95)
	Income			
	Highest	1	1	1
	Quintile 4	1.47 (1.44–1.49)	1.42 (1.38–1.46)	1.33 (1.29–1.36)
	Quintile 3	2.19 (2.15–2.22)	1.91 (1.86–1.97)	1.89 (1.85–1.94)
	Quintile 2	3.47 (3.42–3.52)	2.84 (2.76–2.92)	2.76 (2.70–2.83)
	Lowest	3.12 (3.07–3.17)	3.70 (3.61–3.79)	2.38 (2.33–2.44)
65–74	Gender			
	Female	1	1	1
	Male	1.32 (1.31–1.33)	1.62 (1.59–1.65)	1.41 (1.39–1.43)
	Living alone			
	No	1	1	1
	Yes	1.46 (1.45–1.48)	1.19 (1.16–1.21)	1.48 (1.46–1.50)
	Education			
	Tertiary	1	1	1
	Secondary	1.55 (1.52–1.57)	1.44 (1.40–1.48)	1.56 (1.52–1.59)
	Primary	2.36 (2.32–2.40)	2.13 (2.08–2.18)	2.17 (2.12–2.21)
	Income			
	Highest	1	1	1
	Quintile 4	1.26 (1.23–1.29)	1.37 (1.32–1.42)	1.30 (1.27–1.34)
	Quintile 3	1.60 (1.57–1.64)	1.64 (1.59–1.70)	1.84 (1.80–1.89)
	Quintile 2	2.33 (2.29–2.38)	2.13 (2.06–2.20)	2.42 (2.36–2.48)
	Lowest	2.76 (2.70–2.81)	3.01 (2.91–3.11)	2.11 (2.05–2.16)
75 and over	Gender			
	Female	1	1	1
	Male	1.21 (1.20–1.22)	1.29 (1.27–1.30)	1.29 (1.28–1.30)
	Living alone			
	No	1	1	1
	Yes	1.19 (1.18–1.21)	1.09 (1.07–1.10)	1.20 (1.19–1.21)
	Education			
	Tertiary	1	1	1
	Secondary	1.46 (1.42–1.49)	1.16 (1.13–1.18)	1.29 (1.27–1.31)
	Primary	1.89 (1.84–1.93)	1.45 (1.43–1.48)	1.53 (1.51–1.56)
	Income			
	Highest	1	1	1
	Quintile 4	1.13 (1.10–1.15)	1.07 (1.04–1.10)	1.10 (1.07–1.12)
	Quintile 3	1.29 (1.27–1.32)	1.23 (1.20–1.26)	1.32 (1.29–1.34)
	Quintile 2	1.62 (1.59–1.65)	1.40 (1.37–1.43)	1.43 (1.41–1.46)
	Lowest	1.73 (1.70–1.76)	1.67 (1.63–1.71)	1.26 (1.23–1.29)

Incidence rate ratios (IRRs) of hospitalisations for ambulatory care sensitive conditions (ACSCs) in three Nordic capital areas in 2000–2015 obtained from separately analysed univariate models. Income quintiles were derived from the whole study population

**Fig. 2:**
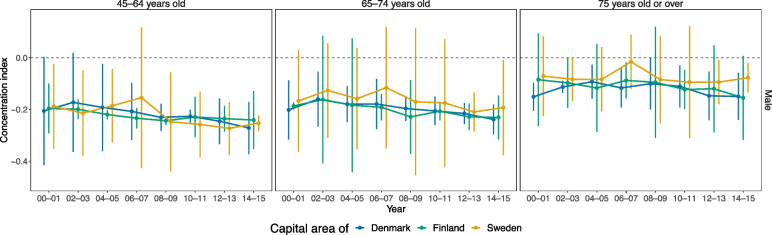
Concentration indices of ACSCs Concentration indices of hospitalisations for ambulatory care sensitive conditions (ACSC) for men in three Nordic capital areas in biennial time-periods in 2000–2015. The results were almost the same for women

The IRRs for individual SEP obtained from univariate models (Table 3) were rather alike between the three capital areas. However, the IRR for living alone seemed lower in Helsinki than elsewhere. This was mainly explained by the low impact of living alone on ACSCs in Finnish women (e.g. these IRRs were 1.12-fold (1.10–1.14) in Finnish women aged 75 and over, and 1.32-fold in Finnish men of respective age) (see also Fig. 1). No similar gender disparity emerged in the Danish and Swedish populations [data not shown]. In each of the three capital areas the incidence of ACSCs emerged with a clear income gradient favouring those in the highest income quintile (most pronounced in those aged 45–64 and least pronounced in those aged 75 and over). However, in Stockholm the IRRs of those in the second lowest quintile seemed higher than of those in the lowest quintile. Over time, income disparities measured with biennial concentration indices seemed rather alike between the three capital areas (Fig. 2). However, while over time these indices in Copenhagen and Helsinki were mainly statistically significantly below value 0 (denoting income-related disparities), those in Stockholm were mainly not – especially in those aged either 65–74 or 75 and over.

IRRs obtained from multilevel models supported the findings of separate univariate models (Table 4). Copenhagen seemed to have the fewest age-related disparities in ACSCs – between the capital areas it had the highest IRRs in those aged 45–64 and the lowest in those aged 75 and over. When calculated from Model 1, the contrasts for the incidence of ACSCs in Copenhagen for those aged 45–64 were 1.40 to 1.71-fold (1.27–1.90) compared to other two capital areas, and 1.22-fold in Helsinki compared to Stockholm. Among those aged 75 and over, contrast in Copenhagen was 0.74-fold (0.67–0.82) compared to Helsinki, and 1.31-fold in Helsinki compared to Stockholm. IRRs for biennial time periods also predicted that over time the decrease of ACSCs was more pronounced in Helsinki than elsewhere. Moreover, when comparing the lowest income quintile to the highest quintile, contrasts for income disparities were the smallest in Stockholm. In Model 2, these contrasts were 1.79-fold (1.75–1.82) in Stockholm and 2.64 to 2.62-fold in other two capital areas – and in Model 4, respectively 1.31-fold (1.27–1.35) and 1.74 to 1.75-fold.

**Table 4:** IRRs of individual factors associated with ACSCs in 2000–2015, multilevel Poisson models

	Model 1	Model 2	Model 3	Model 4
*Predictors*	IRR (CI 95%)	IRR (CI 95%)	IRR (CI 95%)	IRR (CI 95%)
(Intercept)	**0.01 (0.01–0.01)**	**0.01 (0.01–0.01)**	**0.09 (0.08–0.09)**	**0.07 (0.06–0.07)**
Gender [Male]	**1.32 (1.31–1.33)**	**1.42 (1.41–1.42)**	**1.25 (1.24–1.26)**	**1.33 (1.32–1.34)**
Age group [65–74]	**2.93 (2.90–2.96)**	**2.80 (2.77–2.83)**		
Age group [75 and over]	**7.95 (7.88–8.01)**	**6.80 (6.74–6.86)**		
Capital area [Helsinki]	**1.34 (1.25–1.44)**	**1.40 (1.33–1.48)**	**1.49 (1.40–1.59)**	**1.49 (1.41–1.59)**
Capital area [Copenhagen]	**1.67 (1.55–1.81)**	**1.55 (1.47–1.64)**	0.96 (0.89–1.02)	**0.85 (0.80–0.90)**
Time (2-year period)	**0.97 (0.97–0.98)**	**0.98 (0.97–0.98)**	1.00 (0.99–1.01)	1.00 (0.99–1.01)
Capital area [Helsinki]*Age group [65–74]	1.01 (1.00–1.03)	**0.94 (0.92–0.95)**		
Capital area [Helsinki]*Age group [75 and over]	**1.08 (1.06–1.09)**	0.99 (0.97–1.00)		
Capital area [Copenhagen]*Age group [65–74]	**0.85 (0.84–0.86)**	**0.72 (0.71–0.73)**		
Capital area [Copenhagen]*Age group [75 and over]	**0.57 (0.57–0.58)**	**0.50 (0.49–0.51)**		
Capital area [Helsinki]*Time	**0.97 (0.96–0.98)**	**0.97 (0.96–0.98)**	**0.96 (0.95–0.98)**	**0.96 (0.95–0.98)**
Capital area [Copenhagen]*Time	1.01 (1.00–1.02)	1.00 (0.99–1.01)	1.00 (0.99–1.02)	1.00 (0.99–1.02)
Income [4]		**1.27 (1.25–1.29)**		**1.10 (1.08–1.13)**
Income [3]		**1.73 (1.71–1.75)**		**1.36 (1.33–1.39)**
Income [2]		**2.10 (2.08–2.13)**		**1.54 (1.51–1.57)**
Income [Lowest]		**1.79 (1.76–1.81)**		**1.31 (1.28–1.34)**
Capital area [Helsinki]*Income [4]		**1.05 (1.03–1.07)**		0.97 (0.94–1.01)
Capital area [Helsinki]*Income [3]		**0.96 (0.94–0.98)**		**0.91 (0.88–0.94)**
Capital area [Helsinki]*Income [2]		0.98 (0.96–1.00)		**0.93 (0.90–0.96)**
Capital area [Helsinki]*Income [Lowest]		**1.46 (1.43–1.49)**		**1.33 (1.28–1.37)**
Capital area [Copenhagen]*Income [4]		**1.07 (1.05–1.09)**		1.04 (1.00–1.08)
Capital area [Copenhagen]*Income [3]		**1.07 (1.05–1.09)**		0.98 (0.95–1.01)
Capital area [Copenhagen]*Income [2]		**1.24 (1.22–1.26)**		**1.09 (1.05–1.12)**
Capital area [Copenhagen]*Income [Lowest]		**1.48 (1.45–1.51)**		**1.34 (1.30–1.38)**
*Random effects*	σ^2^	σ^2^	σ^2^	σ^2^
Intercept	0.0270	0.0133	0.0203	0.0137
Slope	0.0005	0.0005	0.0007	0.0007

Incidence rate ratios (IRR) of individual factors associated with hospitalisations for ambulatory care sensitive conditions (ACSCs) obtained from multilevel Poisson models in three Nordic capital areas in biennial time periods in 2000–2015 with populations aged + 45 (Models 1–2) and 75 and over (Models 3–4). Each model was adjusted for gender, capital area of residence (Stockholm as reference) and time as well as for selected interactions. Models 1 and 2 were also adjusted for age group and Models 2 and 4 additionally for individual incomes. A bold font indicates statistically significant *P* values (< 0.001)

**Fig. 3:**
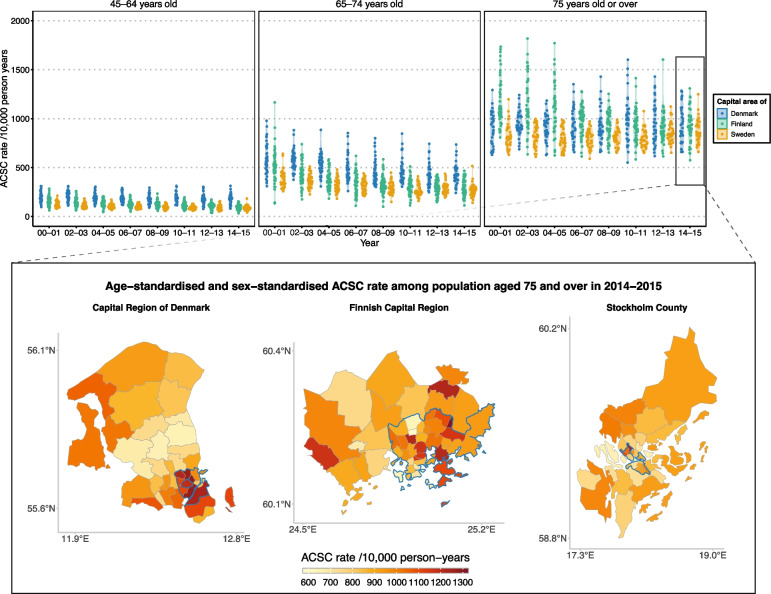
Development of ACSC rates by small areas over time Development of variation in biennial age-standardised and sex-standardised rates of hospitalisations for ambulatory care sensitive conditions (ACSC) by small areas over time in three Nordic capital areas in 2000–2015 among populations aged 45 and over (A) – and geographic distribution of these rates in 2014–15 among populations aged 75 and over (B). Each dot in the violin plot represents ACSC rate for a single small area. Borders of the capital municipalities are highlighted with blue. Adapted and built on municipal, post code and city district divisions downloaded in April 2021 from 1) the Agency for Data Supply and Infrastructure (https://dataforsyningen.dk/data/3559) under the licence of the agency; (https://dataforsyningen.dk/asset/PDF/rettigheder_vilkaar/Vilk%C3%A5r%20for%20brug%20af%20frie%20geografiske%20data.pdf) 2) Helsinki region map (https://hri.fi/data/en_GB/dataset/seutukartta) under the licence Creative Commons Attribution 4.0 (https://creativecommons.org/licenses/by/4.0/); and 3) City of Stockholm (https://dataportalen.stockholm.se/dataportalen/?query=464514632_Dataportalen_EnkelVy_resultset&loc=sv&SplashScreen=no) and Lantmäteriet (https://public.opendatasoft.com/explore/dataset/georef-sweden-kommun/information/?disjunctive.lan_code&disjunctive.lan_name&disjunctive.kom_code&disjunctive.kom_name&sort=year&location=4,62.91317,17.38488&basemap=jawg.light) under the licence Creative Commons CC0 1.0 (https://creativecommons.org/publicdomain/zero/1.0/deed.en)

Geographic disparities over time emerged alongside socioeconomic disparities in each of the three capital areas. Absolute geographic disparities in age-standardised and sex-standardised ACSC rates increased with age (Fig. 3). The figure also shows that the level of these disparities in each age group were always the lowest in Stockholm. Although in 2000 these disparities were the highest in Helsinki, they also seemed to reduce the most over time – eventually reaching the level of Stockholm. On the other hand, in Copenhagen the development of these disparities seemed to stagnate over time.

**Fig. 4:**
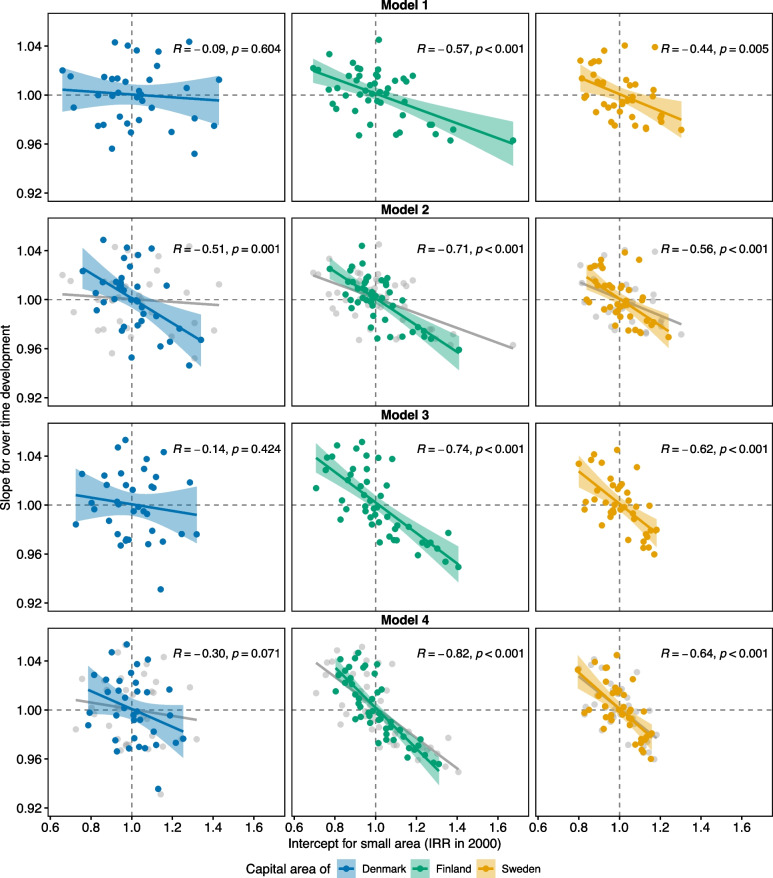
Correlations between small area incidence of ACSCs and their development over time Pearson correlations between estimates obtained from Poisson multilevel models of three Nordic capital areas: 1) small area intercepts, i.e. incidence rate ratios (IRRs) of hospitalisations for ambulatory care sensitive conditions (ACSCs) in 2000 for each small area when compared to the average of capital area they belong to (i.e. x-axis value of 1) – and 2) slopes for over time for the development of incidence of ACSCs in biennial time-periods in 2000–2015 for each small area when compared to the average development in the capital area they belong to (i.e. y-axis value of 1). Each dot represents a single small area. Models 1–2 were analysed with populations aged 45 and over – and Models 3–4 with those aged 75 and over. Each model was adjusted for gender, capital area and time as well as for selected interactions. Models 1 and 2 were also adjusted for age group and Models 2 and 4 for individual incomes (with values not adjusted for incomes presented in grey – i.e. results of Models 1 and 3)

Indeed, while the geographic disparities in Helsinki and Stockholm mainly reduced in the study period, those in Copenhagen persisted (Fig. 4). The figure shows Pearson correlations calculated between two estimates obtained from multilevel models: 1) IRRs of ACSCs in 2000 for each small area when compared to the capital area they belong to, and 2) slopes for development of incidence of ACSCs over time for each small area when compared to the respective average development in their capital area. While IRRs for time were ≤ 1 in each capital area (as shown in Table 4), a small area slope < 1 means that the incidence of ACSCs in that small area decreased more than on average within the capital area – and a slope > 1 that this incidence either decreased less than on average, stagnated or even increased. In both Helsinki and Stockholm, a moderate to strong correlation emerged between small area IRRs > 1 (i.e. incidence of ACSCs higher than on average) and slopes < 1 (i.e. over time this incidence decreased more than on average), and vice versa, between intercepts < 1 and slopes > 1. However, in Copenhagen almost no correlation emerged between these estimates in Models 1 and 3. Although all these correlations were slightly emphasised when the models were adjusted for individual incomes, in Copenhagen a strong and significant correlation emerged in the total study population (Model 2). However, the respective correlation for those aged 75 and over remained non-significant (Model 4).

## Discussion

This register-based study was a first attempt to analyse geographic and socioeconomic disparities over time in ACSCs in three Nordic capital areas – and focused especially on their older adult population. We asked five research questions (RQs), to which the answers are easiest to present by the capital regions. Overall, the level of both ACSCs and their disparities emerged as being rather similar in the three capital areas. Stockholm seemed to have the lowest incidence of ACSCs (RQ1) as well as the most favourable patterns of its mainly decreasing geographic and persisting socioeconomic disparities (RQs 2–4). Its geographic disparities were almost unaffected by individual SEP (RQ5). Although Helsinki initially had the highest level of ACSCs (especially among those aged 75 and over), it also had the fastest decrease in them over time (RQs 1–2). Its geographic disparities decreased, but socioeconomic ones persisted (RQs 3–4). As with Stockholm, individual SEP had little impact on geographic disparities in Helsinki (RQ5). Copenhagen had an average level of ACSCs (RQ1) – and the fewest disparities between age groups, because of a slightly higher incidence of ACSCs among those aged 45–64 than elsewhere, and a low incidence among those aged 75 and over (RQ2). It was the only capital area to have both geographic and socioeconomic disparities that persisted over time (RQs 3–4). Moreover, the observed geographic disparities seemed to partly mirror disparities in the population’s socioeconomic structure between its small areas (i.e. Pearson correlations between small area intercepts and their slopes for development over time were significant only after income adjustment). While the finding for socioeconomic structure emerged in the total study population, it did not emerge when analysed separately in those aged 75 and over (RQ5). Our findings are consistent with earlier studies reporting clear geographic disparities in ACSCs both within [18, 20, 31, 33] and between countries [40, 41] – and that part of these geographic disparities occurred because of differences in individual SEP [18, 20].

### Comments on findings from Copenhagen

Though direct international comparison of incidences of ACSCs might be misleading (as briefly mentioned at the end of the background), some of the findings and differences in ACSC rates between Copenhagen, Helsinki and Stockholm are worth discussing. For Copenhagen, a comparison of these rates shows a rather good, but persistent level of PHC performance among those aged 75 and over. Although ACSC rates due to five chronic conditions have earlier been shown to decrease among the Danish population aged 65 and over [52], with a wider list of ACSCs we only found a limited decrease in ACSC rates among those aged 65–74. One hypothesis is that for those aged 75 and over in Copenhagen the improvements in medical care of these chronic conditions might not have reduced the risk of hospitalisations – possibly because of multimorbidity and frailty. However, we were unable to adjust for individual health status. It is also possible that over time the decrease in ACSCs for chronic conditions in Copenhagen might have been partly substituted by increases in ACSCs for other conditions – especially in the oldest age group. Another hypothesis for the smallest age disparities observed in Copenhagen may be that the Danish PHC has a slightly stronger focus on those aged 75 and over than its Finnish and Swedish counterparts.

While socioeconomic disparities of ACSCs in Copenhagen were similar to those in other Nordic capital areas, over time the persistence of geographic disparities in ACSCs raise concerns that Danish PHC might lack either the tools or incentives to improve its performance. As the administration of the capital area of Copenhagen owns all the local hospitals, it seems unlikely that persisting geographic disparities would mirror different criteria for admitting patients into inpatient hospital care. Indeed, the model for providing PHC in Denmark (i.e. private GPs operating either solo or in small collaborative groups) may limit the feedback that GPs’ receive of their performance in comparison to others [53]. Increasing GPs’ awareness of their local performance could be one way to motivate changes in their practice behaviours [54]. Moreover, geographic disparities in Copenhagen decreased over time only after the models were adjusted using individual incomes – a decrease which emerged in the total study population but not among those aged 75 and over (as shown in Fig. 4). In other words, geographic disparities among those aged 45–74 seemed to partly persist due to PHC in poorly performing areas facing systematic challenges in addressing the care needs of their disadvantaged individuals. One possible interpretation is that this might mirror the accumulation of ill health in disadvantaged areas due to socioeconomic segregation. However, this accumulation does not explain the whole picture as Stockholm seems to have more pronounced level of segregation than Copenhagen [55]. Moreover, further research is needed to decipher which factors associate with persistent geographic disparities among those aged 75 and over. We hypothesise that these findings may relate to both GP workload and remuneration. In Denmark, little geographic variation exists in the number of inhabitants per GPs, without any additional capitation for treating populations with increased need for care (e.g. disadvantaged and/or ageing populations) [2]. Although we analysed only the capital area of Denmark, it is likely that geographic disparities would be found in other parts of Denmark as well [56].

### Comments on findings from Helsinki

Although the observed ACSC rates seem to illustrate improving PHC performance in Helsinki, they also show that Helsinki had room for improvement when compared to its two Nordic counterparts. Our findings of reducing ACSCs over time in Helsinki are congruent with earlier studies for the whole of Finland, in which the decrease in total ACSC rates occurred mainly because of reducing ACSC rates due to chronic conditions [44, 57]. Our findings add that, unlike in Copenhagen and Stockholm, ACSCs in Helsinki have also decreased among those aged 75 and over. On the other hand, this finding co-occurred with excess incidences of ACSCs especially in this age group. While the decrease in ACSCs might seem favourable for older Finns, it was not enough to fully reach the lower incidence rate of ACSCs observed among older Danes and Swedes during our study period. Moreover, these improvements have taken nearly two decades, after which the decrease in ACSC rates in Finland seems to stagnate [58]. While each of the three Nordic countries have reduced their curative hospital bed supply, in Finland these reductions have been the smallest [42]. Thus, we consider it unlikely that the changes in the supply of hospital care were driving the decrease in ACSC rates observed in Helsinki.

Alongside the decreasing incidence of ACSCs we consider that Helsinki has been over time successful in improving geographic equity in PHC performance among its older adults (as shown in Figs. 3 and 4) – even despite the socioeconomic segregation of its small areas. However, among those aged 75 and over the level of geographic disparities in Helsinki seemed to both initially exceed those in the two other Nordic capital areas and reach them only at the end of our study period (Fig. 3). This supports our earlier interpretation of previously untapped potential for improvement within Finnish PHC. The geographic distribution of ACSC rates of small areas in Helsinki followed an earlier reported pattern of socioeconomic segregation [59]. The small areas in which populations have a lower level of education and lower incomes also seemed to have a high incidence of ACSCs, and vice versa. However, we found that small areas with poor PHC performance in 2000 improved more over time than those with good baseline performance. This decrease in geographic disparities in Helsinki emerged before adjustments were made for individual incomes and was only slightly emphasised by it. Moreover, as the capital area of Finland is served by a single provider of hospital care, it is unlikely that the decreasing geographic disparities would mirror differences in either care pathways or hospital admissions criteria.

Finally, the only socioeconomic disparity that clearly differed between the capital area of Finland and the two other Nordic capital areas was between genders living alone. While Finnish men living alone had higher rates than those cohabiting over time, in Finnish women these two rates were nearly similar. This gender disparity supports earlier findings [36]. However, as we found no similar gender disparities in the capital areas of Denmark and Sweden, it is possible that the low risk of ACSCs among Finnish women living alone may be an exception and the overall impact of social isolation on risk of ACSCs might be stronger in other countries.

### Comments on findings from Stockholm

Although the PHC in Stockholm seemed to be performing rather well when compared to Copenhagen and Helsinki, our findings show that its development among older adults has at least stalled over time. Firstly, the incidence of ACSCs in Stockholm emerged either the lowest of the three Nordic capital areas (in those aged 45–74) or joint lowest with Copenhagen (in those aged 75 and over). We did not expect this as the rate of persons aged 65 and over with at least one ACSC in Stockholm stagnates and is among the highest in Sweden [60]. However, it is possible that the low country-level supply of hospital beds in Sweden may limit the incidence of ACSCs for Stockholm in our international comparison [42]. Secondly, our findings for geographic equity in Stockholm were somewhat mixed among those aged 75 and over. The level of geographic variation in this age group seemed low when observed against those of Copenhagen and Helsinki. That is, the level of PHC performance in Stockholm was rather equitable between its small areas. However, although Fig. 4 suggests a converging trend in the incidence of ACSCs between small areas over time, Fig. 3 shows an initially declining but then slowly increasing variation of biennial ACSC rates especially among those aged 75 and over. It seems that this possible increase might have begun after 2008, thus coinciding with the local implementation of the national Free Choice in PHC reform. While this reform gave patients free choice over their PHC provider, it also opened the Swedish tax-funded health system up to market-based competition [61, 62]. Simultaneously Stockholm adopted a mainly fee-for-services payment model for reimbursing its PHC providers. Since the implementation of these changes, the access to PHC in Stockholm has improved to a degree in favour of those with low incomes [63]. However, at the same time geographic equity in this access has slightly declined [61]. It is possible that the declining geographic equity in access to PHC might have also translated into a slight increase in geographic disparities in PHC performance observed at the end of our study period. This interpretation is in line with the finding that since the reform, new PHC facilities in Sweden have been established mainly in areas with a low proportion of older adults living alone [64]. However, analysis of more recent data is needed.

Though the findings for geographic equity in Stockholm were slightly mixed, the findings for socioeconomic disparities seemed partly favourable for Stockholm. The incidence of ACSC in the lowest income quintile in Stockholm was somewhat lower than in the respective quintile in Copenhagen and Helsinki. This differs from the smooth income gradient commonly observed [18, 38], and might also be the reason for mainly non-significant concentration indices in Stockholm. It might be that the Swedish PHC reaches those in the lowest income quintile more efficiently than its Danish or Finnish counterparts. Indeed, access to PHC among the adult population in Stockholm favours those on low incomes [63], while in Denmark this access seems rather equitable [65] and in Finland it slightly favours those on high incomes (due to private and occupational health care) [66]. Moreover, adjusting for income had almost no impact on geographic disparities among older adults – as shown in Fig. 4. This finding suggests that over time the Swedish PHC system seems successful in addressing the possible ill-effects of socioeconomic segregation on PHC performance.

### Policy recommendations and lessons learnt

In the light of our findings, we encourage Danish policymakers to request a more thorough assessment of how their PHC performs and how the care is coordinated between the different parts of the service system – especially with regard to assess social inequality of areas with more or less disadvantaged populations. For Finnish policymakers our findings emphasise that the promising decrease in ACSC has so far only brought PHC performance in Helsinki close to the level of its two Nordic counterparts. Thus, strengthening PHC should be further encouraged to maintain this promising development. In addition, we encourage Swedish policymakers to request a national analysis with more recent data to decipher whether the decreasing trend of geographic disparities of PHC performance might have turned into increasing one after the national Free Choice in PHC reform.

### Strengths and limitations

In this study, we were able to apply both administrative small areas and the effects of individual SEP in an international comparison of ACSCs, which represents an improvement on respective earlier comparisons that have mainly applied to municipalities or regions as well as area-level SEP [40, 41]. However, we assessed geographic variation in ACSCs within three capital areas (urban settings), which might differ from the general (nationwide) context. In addition, we were able to describe socioeconomic disparities through multiple measures of individual SEP, as suggested in earlier studies [67, 68]. However, international comparison of ACSCs can yield additional bias when compared to an analysis of ACSCs within a single country. For example, the low incidence of ACSCs in Stockholm might not only mirror good PHC performance but also low national supply of hospital beds. It is also possible that variables for SEP may not be directly comparable between countries as there might exist differences in either the compilation of register data or the composition of subgroups formed with a common definition of SEP. Moreover, the classifications of diagnoses and procedures used to obtain ACSCs might differ between countries and the national contexts of health care (e.g. care pathways from PHC to hospital care and criteria for inpatient care) might confound the analysis. To diminish the possibility of bias related to classifications, we applied Nordic conversion tables for diagnoses and procedures (available from www.nordcase.org) to adapt the ACSC definition to national classifications (see also Additional file 1). Moreover, the Nordic register data for individual hospitalisations has been found to be of good quality [69–71]. Our findings and interpretations are specific to the applied definition of ACSCs, and usage of a different list of conditions would have likely impacted both the observed levels and variations of ACSCs [72]. We also consider that our study approach of assessing development of geographic and socioeconomic disparities over time (rather than simple direct comparison of ACSC rates) between the three Nordic capital areas diminishes the possible impacts and confounding effects of different national contexts.

We acknowledge that ACSC has some weaknesses as an indicator of PHC quality – mainly because it is also impacted by factors confounding the measurement of PHC performance or beyond the scope of PHC (as described in the Background), which we were unable to include in the analysis. For example, this study is limited by the lack of variables for individual health status, which would likely explain some geographic disparities in ACSCs [18, 20]. To prevent indirect identification of individuals in small areas with just a few annual ACSCs, we had to refrain from both dividing ACSC into three subgroups and including multiple measures of SEP into the multilevel models. This study includes several models in univariate and multivariate multilevel context, which are followed by multiple testing of hypotheses. Individual tests were unadjusted for multiple testing in this study, and thus, should be interpreted with caution. It is also possible, that our analysis (with no upper age-limit) of the effects of SEP on ACSCs might be somewhat affected either by earlier rather than present SEP, or by selective survival [73–75]. However, in the light of earlier literature, at least the effect of selective survival is likely to be minor [74].

## Conclusions

This study shows that PHC performance and related socioeconomic equity have over time developed rather similarly among the older adults in the capital areas of Denmark, Finland and Sweden. This likely reflects the rather similar health care systems and sociodemographic structure of populations in these capital areas. However, while geographic equity in PHC performance over time improved in the capital areas of Finland and Sweden, in the capital area of Denmark it clearly persisted. This might mirror that patient volume and the remuneration model of Danish GPs may fail to take the increasing local workload within disadvantaged areas fully into account. Moreover, while the Finnish PHC emerged as an underdog in the early 2000s, it has been able to improve its performance over time to almost reach the respective levels of the Danish and the Swedish PHCs. And finally, while PHC performance emerged rather favourably for the capital area of Sweden, our findings raise concerns for its possible declining geographic equity after the implementation of Swedish free choice reform.

### Supplementary Information

**Additional file 1: **Definition table of hospitalisations for ambulatory care sensitive conditions (ACSC).

**Additional file 2: **Definition of analysed small areas.

## Data Availability

As the data in this study consists of sensitive individual data, it is not publicly available due to national data protection legislation and GDPR. Permissions to use the register data for research purposes can be obtained from the competent register authorities: Finnish Institute for Health and Welfare, Statistics Denmark, Statistics Finland, Statistics Sweden, and Region Stockholm.

## Abbreviations

ACSC: Hospitalisations for ambulatory care sensitive conditions
C: Concentration indices
GP: General practitioner
ICD: International Statistical Classification of Diseases
IRR: Incidence rate ratio
LISA: Longitudinal integrated database for health insurance and labour market studies
NOMESCO: Nordic Medico-Statistical Committee
PHC: Primary health care
RQ: Research question
SEP: Socioeconomic position
