# Supplementary material for: Performing up to Nordic principles? Geographic and socioeconomic equity in ambulatory care sensitive conditions among older adults in capital areas of Denmark, Finland and Sweden in 2000–2015
Source: BMC Health Serv Res. 2023 Aug 7;23:835. doi: 10.1186/s12913-023-09855-0 (PMC10405465; doi:10.1186/s12913-023-09855-0)
Supplement: Supplementary file 1 — Additional file 1: Definition table of hospitalisations for ambulatory care sensitive conditions (ACSC). [file 12913_2023_9855_MOESM1_ESM.pdf]

Additional file 1. Definition table of hospitalisations for ambulatory care sensitive conditions (ACSC).

ACSC conditions	*ICD-9 (Finland)*	*ICD-9 (Sweden)*	*ICD-10*	*Definition notes*
Vaccine-preventable				
Bacterial Pneumonia & Influenza	481, 4822A, 4823A, 4824A, 4829X, 483, 485, 487	481, 4822, 4823, 4829, 483, 486, 487	J09, J10, J11, J13, J14, J15.3, J15.4, J15.7, J15.9, J16.8, J18.1, J18.8, J18.9	In any diagnosis field, do not accept if D57 is as a secondary diagnosis
Immunization-Related and Preventable Conditions	032, 033, 037, 045, 055, 056, 0703A, 072, 3200A	032, 033, 037, 045, 055, 056, 0703, 072, 3200, 3232A	A35, A36, A37, A80, B05, B06, B16.1, B16.9, B18.0, B18.1, B26, G00.0, M01.4	In any diagnosis field
Chronic				
Acute Bronchitis	4660A	4660	J20	Only when primary diagnosis, and J41-J44 or J47 as a secondary diagnosis
Angina	4110, 4111, 413	411, 413	I20, I24.0, I24.8, I24.9	Primary diagnosis only, exclude cases with surgical procedures (A)
Asthma	493	493	J45, J46	Primary diagnosis only
COPD	491, 492, 494, 496	491, 492, 494, 496	J41, J42, J43, J44, J47	Primary diagnosis only
Congestive Heart Failure	4029B, 428	428	I11.0, I50, J81	Primary diagnosis only, exclude cases with cardiac procedures (C)
Diabetes Complications	2501-2508	2501-2509	E10.0-10.8, E11.0-E11.8, E12.0-E12.8, E13.0-E13.8, E14.0-E14.8	Primary diagnosis only
Hypertension	401, 4029A	401, 402	I10, I11.9	Primary diagnosis only, exclude cases with cardiac procedures (C)
Iron Deficiency Anaemia	280	280	D50.1-D50.9	Primary diagnosis only
Nutritional Deficiencies	260, 261, 262, 2680A, 2681A	260, 261, 262, 2680, 2681	E40, E41, E42, E43, E55.0, E64.3	Primary diagnosis only
Acute				
Cellulitis	681, 682, 683, 6860A-6868C	681, 682, 683, 6860-6861	L03, L04, L08.0, L08.8, L88, L98.0	Primary diagnosis only, exclude cases with other surgical procedures than skin procedures (B)
Convulsions and Epilepsy	345, 6426, 7803A	345, 6426, 7803	G40, G41, O15, R56	Primary diagnosis only
Dehydration and Gastroenteritis	2765A, 5583A, 5589X	2765, 558	E86, K52.2, K52.8, K52.9	Primary diagnosis only
Dental Conditions	101, 521, 522, 523, 525, 528	101, 521, 522, 523, 525, 528	A69.0, K02-K06, K08, K09.8, K09.9, K12, K13	Primary diagnosis only
Gangrene	7854A+2506	7854 + 2507	R02	In any diagnosis field
Kidney and Urinary Tract Infections	5900A, 5901A	590, 5901	N10, N11, N12, N13.6	Primary diagnosis only
Pelvic Inflammatory Disease	614	614, 6150A, 6151A, 6159A, 6160A	N70, N73, N74	Primary diagnosis only
Perforated or Bleeding Ulcer	5310B-5310C, 5311B-5311C, 5312B-5312C, 5313B-5313C, 5314B-5314C, 5315B-5315C, 5320B-5230C, 5321B-5321C, 5322B-5322C, 5323B-5323C, 5324B-5324C, 5325B-5325C, 5340B-5340C, 5341B-5341C, 5348B-5348C	5310-5312, 5314-5316, 5320-5322, 5324-5326, 5330-5332, 5334-5336, 5340-5342, 5344-5346	K25.0-K25.2, K25.4-K25.6, K26.0-K26.2, K26.4-K26.6, K27.0-K27.2, K27.4-K27.6, K28.0-K28.2, K28.4-K28.6	Primary diagnosis only
Severe ENT infection	0340, 382, 462, 463, 465, 4721A	0340, 3810A, 382, 462, 463, 465, 4721	H66, H67, J02, J03, J06, J31.2	Primary diagnosis only

COPD = chronic obstructive pulmonary disease; ENT = ear, nose and throat; ICD-9 = International Statistical Classification of Diseases, 9th revision; ICD-10 = International Statistical Classification of Diseases, 10th revision

A) Angina, no procedure field contains any of the following:

Denmark
KA-KZ

Finland
AA4WT, AA400, AA5LT, AAA-AAW, AB4AT, AB4BT, AB4CT, AB4DT, AB4FT, AB5, AB6AT, AB6BT, AB6CT, AB6DT, ABA-ABW, ACA-ACW, AD-AW, AXX90, BA3AT, BA4KT, BAA, BB2AT, BB3AT, BB4WT, BBA, BC1LT, BC2-BC4, BCA, BD-BW, CAA-CAW, CB, CCA-CCW, CD-CJ, CKA-CKW, CW, DAA-DAW, DB, DCA-DCW, DD, DEA-DEW, DFA-DFE, DFW99, DG, DHA-DLW, DMA-DPW, DQ-DW, EA, EBA-EBW, EC, EDA-EDW, EEA-EW, EF, EGA-EGW, EHA-EHW, EJ-EK, EL3AT, EL3RT, EL3YT, EL4 ,ELA-ELW, EMA-EMW, EP1AT, EP1LT, EP2AT, EP3AT, ENA-ENW, EW, FA, FBA-FBW, FCA-FCE, FD, FE1AT, FE2, FEA-FEW, FF-FL, FMA-FMW, FN1ST, FN1XT, FNA-FNW, FP-FX, GA2-GA4, GAA-GAW, GBA-GBW, GCA, GD1AT, GD1BT, GD1CT, GD1LT, GD2AT, GD2BT, GD2CT, GD3, GDA-GDW, GE1AT, GE1CT, GE1DT, GE2, GEA-GEW, GW, HA0, HA1AT, HA1DT, HA1MT, HA1ST, HA2-HA5, HAA-HAF, HW, JA1LT, JA2-JA3, JAA-JAW, JB, JCA-JCW, JDA-JDW, JE, JF3, JFA-JFW, JGA-JGW, JHA-JHW, JJ1AT, JJ2-JJ8, JJA-JJW, JK1-JK2, J3KAT, JK3BT, JK3CT, JK3FT, JK3LT, JK3NT, JK3RT, JK4-JK5, JKA-JKW, JL1-JL3, JLA-JLW, JM1AT, JM1LT, JM2, JMA-JMW, JN4LT, JW, JX1LT, JX1RT, JXA, KA2AT, KA3AT, KA3CT, KA3DT, KA3LT, KA4-KA6, KAA-KAW, KBA-KBW, KC1AT, KC2AT, KC3AT, KCA-KCW, KDA-KDW, KE1AT, KE1CT, KE2, KEA-KEW, KF1-KF7, KF8AT, KF8KT, KFA-KFW, KGA-KGW, KH1AT, KH1BT, KH1CT, KH1CT, KH1DT, KH1FT, KH1YT, KKA-KKW, KW, KX, LA1, LAA-LAW, LB1AT, LB1YT, LBA-LBW, LCA-LCW, LDA-LDW, LEA-LEW, LF-LW, LX1LT, MAA-MAW, MBA-MBW, MC-MW, NA0, NA6CT, NA7BT, NA7FT, NA7KT, NA7LT, NA9KT, NAA-NAW, NB1AT,NB1BT, NB1ZT, NB2, NBA-NBW, NCA-NCW, NDA-NDW, NEA-NEW, NFA-NFW, NGA-NGW, NHA-NHW, NJ3LT, NK1AT, NK1CT, NK1DT, NK1LT, NK2-NK3, NK4AT, NK4BT, NK5, NK6AT, NK6BT, NK6CT, NK6DT, NK6KT, NK7AT, NX, PA2ZT, PA3-PA5, PA6AT, PA8KT, PA9KT, PAA-PAW, PB1AT, PB1BT, PB1ST, PB1YT, PBA-PBW, PC2DT, PC2ET, PC5AT, PC5BT, PC5DT, PC5ET, PC5GT, PC5HT, PC5JT, PC5NT, PC5PT, PC5YT, PC6DT, PC6ET, PC6FT, PC7NT, PCA-PCW, PD1AT, PD1YT, PD2DT, PD3, PD4ST, PD5YT, PD6YT, PD7YT, PDA-PDW, PE, PG1AT, PG1BT, PG1ET-PG1LT, PG1MT-PG1VT, PG1YT-PG3YT, PG5RT-PG6NT, PGA-PGW, PH1AT, PH1FT, PH1UT, PH2ST, PH3YT, PH4AT, PH5GT, PH6GT, PH7FT, PH7UT, PH9AT, PH9ST, PH900, PHA-PHW, PJ2AT, PJ2CT, PJ2HT, PJ3-PJ4, PJ5AN, PJA-PJW, PW, PXA-PXX, QAA-QAW, QBA-QBW, QCA-QCW, QDA-QDW, QW, QXA-QXW, QX2ZT, QX3AT, QX3CT, QX3LT, QX3YT, QX4, S, TAA-TAD, TAW99, TA100, TBA-TJF, TJG10, TJJ, TJL-TJW, TK-TL, TMA-TPX, TQ, U, WXQ, WW20, WW30-WW31, WW40, WW50, WX100-WX105, WX140-WX144, WX7-WX9, XCC00, XFE00, XFN96, XFX00, XFX10, XFX20, XFX97, XJW99, XPX00, XPX04, XPX08, XPX99, XW000, XW1-XW5, XX1AT, XX1BT, XX1CT, XX1DT, XX1XT, XX2AT-XX2DT, XX2XT, XX3AT-XX3DT, XX3XT, XX4-XX7, Y, ZC-ZP, ZS-ZX, ZZ

Sweden
AA019, AA064, AA081-AA082, AA090, AA095-AA096, AAA-AAW, ABA-ABW, ACA-ACW, ADA-ADW, AEA-AEW, AJ023, AJ025, AK060-AK063, AP029, AP051-AP052, AP066, AP075, AR023, AR025, AV014, AV057, AV122, AV127, AW, B, C, DA, DBA-DBW, DC, DD002-DD006, DD008, DD011, DD016-DD019, DDA-DDW, DE-DH, DJ003-DJ009, DJ011-DJ026, DJA-DJW, DK-DM, DN001-DN005, DN010, DNA-DNW, DP-DT, DV002-DV003, DV005-DV008, DV011-DV023, DV025-DV030, DV034-DV035, DV037-DV068, DV70-DV072, DV074-DV075, DV079, DV082-DV083, DV090-DV092, DV095-DV096, DV144-DV145, DW, E, F, GA001, GA002-GA012, GA014-GA016, GAA-GAW, GBA-GBW, GCA, GDA-GDW, GE-GW, HAA-HAF, HW, J, K, L, M, N, PAA-PAW, PBA-PBW, PCA-PCW, PDA-PDW, PEA-PEW, PFA-PFW, PGA-PGW, PHA-PHW, PJA-PJW, PW-PX, QAA-QAW, QBA-QBW, QCA-QCW, QD002, QDA-QDW, QW, QXA-QXC, SA-SR, SS104-SS106, SS4, SS599, SS799, SS899, SX, TA-TH, TJA-TJF, TJG10, TJJ-TJW, TK-TQ, U, XA, XCC00, XF, XJW, XPX, XV009-XV010, Y, ZC-ZP, ZS-ZU, ZX-ZZ

B) Cellulitis, no procedure field contains any of the following:

Denmark

KA-KP, KQAA00, KQAA60, KQAA99, KQAB00-KQAB05, KQAB99, KQAC, KQAD20, KQAE-KQAF, KQAG10-KQAG99, KQAJ, KQBA00, KQBA99, KQBB00-KQBB05, KQBB99, KQBC, KQBD20, KQBE, KQBG10-KQBG99, KQBJ, KQCA00, KQCA30, KQCA99, KQCB00-KQCB05, KQCB99, KQCC, KQCD20, KQCE-KQCG, KQCJ, KQDA00, KQDA99, KQDB00-KQDB05, KQDB99, KQDC, KQDD20, KQDE, KQDG10-KQDG99, KQDJ, KQXA00, KQXA99, KQXB00-KQXB05, KQXB99, KQXC, KQXD20, KQXE, KQXG10-KQXG99, KQXJ, KTA-KTP, KTQX00-KTQX10, KTX, KU, KY, KZ

Finland

AA4WT, AA400, AA5LT, AAA-AAW, AB4AT, AB4BT, AB4CT, AB4DT, AB4FT, AB5, AB6AT, AB6BT, AB6CT, AB6DT, ABA-ABW, ACA-ACW, AD-AW, AXX90, BA3AT, BA4KT, BAA, BB2AT, BB3AT, BB4WT, BBA, BC1LT, BC2-BC4, BCA, BD-BW, CAA-CAW, CB, CCA-CCW, CD-CJ, CKA-CKW, CW, DAA-DAW, DB, DCA-DCW, DD, DEA-DEW, DFA-DFE, DFW99, DG, DHA-DLW, DMA-DPW, DQ-DW, EA, EBA-EBW, EC, EDA-EDW, EEA-EW, EF, EGA-EGW, EHA-EHW, EJ-EK, EL3AT, EL3RT, EL3YT, EL4 ,ELA-ELW, EMA-EMW, EP1AT, EP1LT, EP2AT, EP3AT, ENA-ENW, EW, FA, FBA-FBW, FCA-FCE, FD, FE1AT, FE2, FEA-FEW, FF-FL, FMA-FMW, FN1ST, FN1XT, FNA-FNW, FP-FX, GA2-GA4, GAA-GAW, GBA-GBW, GCA, GD1AT, GD1BT, GD1CT, GD1LT, GD2AT, GD2BT, GD2CT, GD3, GDA-GDW, GE1AT, GE1CT, GE1DT, GE2, GEA-GEW, GW, HA0, HA1AT, HA1DT, HA1MT, HA1ST, HA2-HA5, HAA-HAF, HW, JA1LT, JA2-JA3, JAA-JAW, JB, JCA-JCW, JDA-JDW, JE, JF3, JFA-JFW, JGA-JGW, JHA-JHW, JJ1AT, JJ2-JJ8, JJA-JJW, JK1-JK2, J3KAT, JK3BT, JK3CT, JK3FT, JK3LT, JK3NT, JK3RT, JK4-JK5, JKA-JKW, JL1-JL3, JLA-JLW, JM1AT, JM1LT, JM2, JMA-JMW, JN4LT, JW, JX1LT, JX1RT, JXA, KA2AT, KA3AT, KA3CT, KA3DT, KA3LT, KA4-KA6, KAA-KAW, KBA-KBW, KC1AT, KC2AT, KC3AT, KCA-KCW, KDA-KDW, KE1AT, KE1CT, KE2, KEA-KEW, KF1-KF7, KF8AT, KF8KT, KFA-KFW, KGA-KGW, KH1AT, KH1BT, KH1CT, KH1CT, KH1DT, KH1FT, KH1YT, KKA-KKW, KW, KX, LA1, LAA-LAW, LB1AT, LB1YT, LBA-LBW, LCA-LCW, LDA-LDW, LEA-LEW, LF-LW, LX1LT, MAA-MAW, MBA-MBW, MC-MW, NA0, NA6CT, NA7BT, NA7FT, NA7KT, NA7LT, NA9KT, NAA-NAW, NB1AT,NB1BT, NB1ZT, NB2, NBA-NBW, NCA-NCW, NDA-NDW, NEA-NEW, NFA-NFW, NGA-NGW, NHA-NHW, NJ3LT, NK1AT, NK1CT, NK1DT, NK1LT, NK2-NK3, NK4AT, NK4BT, NK5, NK6AT, NK6BT, NK6CT, NK6DT, NK6KT, NK7AT, NX, PA2ZT, PA3-PA5, PA6AT, PA8KT, PA9KT, PAA-PAW, PB1AT, PB1BT, PB1ST, PB1YT, PBA-PBW, PC2DT, PC2ET, PC5AT, PC5BT, PC5DT, PC5ET, PC5GT, PC5HT, PC5JT, PC5NT, PC5PT, PC5YT, PC6DT, PC6ET, PC6FT, PC7NT, PCA-PCW, PD1AT, PD1YT, PD2DT, PD3, PD4ST, PD5YT, PD6YT, PD7YT, PDA-PDW, PE, PG1AT, PG1BT, PG1ET-PG1LT, PG1MT-PG1VT, PG1YT-PG3YT, PG5RT-PG6NT, PGA-PGW, PH1AT, PH1FT, PH1UT, PH2ST, PH3YT, PH4AT, PH5GT, PH6GT, PH7FT, PH7UT, PH9AT, PH9ST, PH900, PHA-PHW, PJ2AT, PJ2CT, PJ2HT, PJ3-PJ4, PJ5AN, PJA-PJW, PW, PXA-PXX, QAA25, QAB00-QAB05, QAB99, QAC, QAD20, QAE-QAF, QAG10-QAG99, QAJ, QBA25, QBB00-QBB05, QBB99, QBC, QBD20, QBE, QBG10-QBG99, QBJ, QCA25, QCA30, QCB00-QCB05, QCB99, QCC, QCD20, QCE-QCG, QCJ, QDA25, QDB00-QDB05, QDB99, QDC, QDD20, QDE, QDG10-QDG99, QDJ, QXA25, QXB00-QXB05, QXB99, QXC, QXD20, QXE, QXG10-QXG99, QXJ, QX2ZT, QX3AT, QX3CT, QX3LT, QX3YT, QX4, S, TAA-TAD, TAW99, TA100, TBA-TJF, TJG10, TJJ, TJL-TJW, TK-TL, TMA-TPX, TQA-TQD, TQW00, TQW02, TQW30-40, TQW99, TQX00-TQX10, U, WW20, WW30-WW31, WW40, WW50, WX100-WX105, WX140-WX144, WX7-WX9, XCC00, XFE00, XFN96, XFX00, XFX10, XFX20, XFX97, XJW99, XPX00, XPX04, XPX08, XPX99, XW000, XW1-XW5, XX1AT, XX1BT, XX1CT, XX1DT, XX1XT, XX2AT-XX2DT, XX2XT, XX3AT-XX3DT, XX3XT, XX4-XX7, Y, ZC-ZP, ZS-ZX, ZZ

Sweden

AA019, AA064, AA081-AA082, AA090, AA095-AA096, AAA-AAW, ABA-ABW, ACA-ACW, ADA-ADW, AEA-AEW, AJ023, AJ025, AK060-AK063, AP029, AP051-AP052, AP066, AP075, AR023, AR025, AV014, AV057, AV122, AV127, AW, B, C, DA, DBA-DBW, DC, DD002-DD006, DD008, DD011, DD016-DD019, DDA-DDW, DE-DH, DJ003-DJ009, DJ011-DJ026, DJA-DJW, DK-DM, DN001-DN005, DN010, DNA-DNW, DP, DQ019, DQA-DQW, DT, DV002-003, DV005-DV008, DV011-DV023, DV025-DV30, DV034-DV035, DV037-DV041, DV043-DV046, DV50-DV068, DV70-DV072, DV074-DV075, DV079, DV082-DV083, DV090-DV092, DV095-DV096, DV144-DV145, DW, E, F, GA001, GA002-GA012, GA014-GA016, GAA-GAW, GBA-GBW, GCA, GDA-GDW, GE-GW, HAA-HAF, HW, J, K, L, M, N, PAA-PAW, PBA-PBW, PCA-PCW, PDA-PDW, PEA-PEW, PFA-PFW, PGA-PGW, PHA-PHW, PJA-PJW, PW-PX, QAB00-QAB05, QAB99, QAC, QAD20, QAE-QAJ, QBB00-QBB05, QBB99, QBC, QBD20, QBE, QBG10-QBG99, QBJ, QCB00-QCB05, QCB99, QCC, QCD20, QCE-QCG, QCJ, QDB00-QDB05, QDB99, QDC, QDD20, QDE, QDG10-QDG99, QDJ, QXB05, QXB99, QXC, SA-SR, SS104-SS106, SS4, SS599, SS799, SS899, SX, TA-TH, TJA-TJF, TJG10, TJJ-TJW, TK-TQ, U, XA, XCC00, XF, XJW, XPX, XV009-XV010, Y, ZC-ZP, ZS-ZU, ZX-ZZ

C) Congestive heart failure and hypertension, no procedure field contains any of the following:

Denmark
KFE, KFFA00, KFFA10-KFFA30, KFFA96, KFFB-KFFW, KFG, KFH, KFJA00, KFJA96, KFJB-KFJW, KFK, KFLA00, KFLA96, KFLB-KFLW, KFM-KFN, KFPA-KFPF, KFPH-KFPW, KFQ, KFXA-KFXN, KZFX03, KZFX7

Finland
FEA-FEW, FFA00, FFA10-FFA30, FFA96, FFB-FFW, FG-FH, FJA00, FJA96, FJB-FJW, FKA-FKW, FK1BT, FLA00, FLA96, FLB-FLW, FMA-FMW, FN1AT, FN1BT, FN1ST, FN1XT, FN1YT, FNA-FNW, FPA-FPF, FPH-FPW, FQ, FXA00-FXN00, TFN10, TFN99, TFP00, TFP40-TFP59

Sweden
DF001-DF005, DF013, DF019-DF024, FEA-FEW, FFA00, FFA10-FFA30, FFA96, FFB-FFW, FG-FH, FJA00, FJA96, FJB-FJW, FK, FLA00, FLA96, FLB-FLW, FM-FN, FPA-FPF, FPH-FPW, FQ, FXA00-FXN00, TFP00
