# Supplementary material for: Performing up to Nordic principles? Geographic and socioeconomic equity in ambulatory care sensitive conditions among older adults in capital areas of Denmark, Finland and Sweden in 2000–2015
Source: BMC Health Serv Res. 2023 Aug 7;23:835. doi: 10.1186/s12913-023-09855-0 (PMC10405465; doi:10.1186/s12913-023-09855-0)
Supplement: Supplementary file 2 — Additional file 2: Definition of analysed small areas. [file 12913_2023_9855_MOESM2_ESM.pdf]

Additional file 2. Definition of analysed small areas.

Capital Region of Denmark
Small areas (n=36)

Municipality	*Code*
Frederiksberg	0147
Ballerup	0151
Brøndby	0153
Dragør	0155
Gentofte	0157
Gladsaxe	0159
Glostrup	0161
Herlev	0163
Albertslund	0165
Hvidovre	0167
Høje–Taastrup	0169
Lyngby–Taarbæk	0173
Rødovre	0175
Ishøj	0183
Tårnby	0185
Vallensbæk	0187
Furesø	0190
Allerød	0201
Fredensborg	0210
Helsingør	0217
Hillerød	0219
Hørsholm	0223
Rudersdal	0230
Egedal	0240
Frederikssund	0250
Halsnæs	0260
Gribskov	0270

Municipality	*Code*	*District*	*Postal code*
Copenhagen	0101	Indre by	1050–1074, 1100–1107, 1110–1131, 1150–1162, 1164–1175, 1200–1216, 1218–1221, 1250–1261, 1263–1268, 1270–1271, 1300–1304, 1306–1329, 1350, 1352–1371, 1400–1403, 1406–1441, 1450–1468, 1470–1473, 1550–1560, 1562–1564, 1567–1569, 1571–1576, 1600–1609, 1611–1614

		Østerbro	2100, 2150, 2900
		Nørrebro	1631–1635, 2200
		Vesterbro/Kongens Enghave	1561, 1570, 1577, 1610, 1615–1624, 1650–1677, 1700–1712, 1714–1739, 1748–1766, 1770–1775, 1777, 1799, 1810, 2450
		Valby	2000, 2500, 2610, 2650
		Vanløse	2720
		Brønshøj–Husum	2700, 2730
		Bispebjerg	2400, 2860
		Amager Øst	2300
		Amager Vest	2770

Finnish Capital Region
Small areas (n=47)

Municipality	*Code*	*Major district*	*District code*
Espoo	049	Suur–Leppävaara	1
		Suur–Tapiola	2
		Suur–Matinkylä	3
		Suur–Espoonlahti	4
		Suur–Kauklahti	5
		Vanha–Espoo	6
		Pohjois–Espoo	7
Vantaa	092	Myyrmäki	1
		Kivistö	2
		Aviapolis	3
		Tikkurila	4
		Koivukylä	5
		Korso	6
		Hakunila	7
Kauniainen	235		

Municipality	*Code*	*Minor district*	*District code*
Helsinki	091	Vironniemi	101
		Ullanlinna	102
		Kampinmalmi	103
		Taka–Töölö	104
		Lauttasaari	105
		Reijola	201
		Munkkiniemi	202
		Haaga	203

		Pitäjänmäki	204
		Kaarela	205
		Kallio	301
		Alppiharju	302*
		Vallila	303
		Pasila	304
		Vanhakaupunki	305
		Maunula	401
		Länsi–Pakila	402
		Tuomarinkylä	403
		Oulunkylä	404
		Itä–Pakila	405
		Latokartano	501
		Pukinmäki	502
		Malmi	503
		Suutarila	504
		Puistola	505
		Jakomäki	506
		Kulosaari	601
		Herttoniemi	602
		Laajasalo	603
		Vartiokylä	701
		Myllypuro	702
		Mellunkylä	703
		Vuosaari	704
		Östersundom	801**

*Alppiharju combined to Vallila, **Östersundom combined to Vuosaari

Region Stockholm

Small areas (n=39)

Municipality	*Code*
Upplands Väsby	0114
Vallentuna	0115
Österåker	0117
Värmdö	0120
Järfälla	0123
Ekerö	0125
Huddinge	0126
Botkyrka	0127

Salem	0128
Haninge	0136
Tyresö	0138
Upplands Bro	0139
Nykvarn	0140
Täby	0160
Danderyd	0162
Sollentuna	0163
Södertälje	0181
Nacka	0182
Sundbyberg	0183
Solna	0184
Lidingö	0186
Vaxholm	0187
Norrtälje	0188
Sigtuna	0191
Nynäshamn	0192

Municipality	*Code*	*District*	*District code*
Stockholm	0180	Spånga–Tensta	80103
		Hässelby–Vällingby	80104
		Bromma	80106
		Kungsholmen	80108
		Norrmalm	80109
		Östermalm	80110
		Skarpnäck	80115
		Farsta	80118
		Älvsjö	80121
		Skärholmen	80124
		Rinkeby–Kista	80125
		Hägersten	80126
		Södermalm	80127
		Enskede–Årsta–Vantör	80128
